# 0435. Pressure-support ventilation compared to pressure-controlled ventilation in experimental emphysema

**DOI:** 10.1186/2197-425X-2-S1-P27

**Published:** 2014-09-26

**Authors:** GA Padilha, I Henriques, L Moraes, MV Oliveira, IP Ramos, PJ Miranda, LF Horta, RC Goldenberg, P Pelosi, PL Silva, PRM Rocco

**Affiliations:** Federal University of Rio de Janeiro, Carlos Chagas Filho Biophysics Institute, Rio deJaneiro, Brazil; University of Genoa, Department of Surgical Sciences and Integrated Diagnostics, Genoa, Italy

## Introduction

Emphysema is characterized by irreversible enlargement of the airspaces distal to the terminal bronchiole, accompanied by destruction of their walls, and impairment of cardiac function. Acute exacerbation of emphysema leads to increased morbidity, mortality and, in some cases, requirement of invasive mechanical ventilation (MV). So far, no study has compared the impact of pressure-controlled ventilation (PCV) and pressure-support ventilation (PSV) on lung mechanics and histology as well as cardiac function in experimental emphysema.

## Objectives

Our aims were

1. to develop an elastase-induced emphysema model in rats that results in lung mechanical and histological impairment and *cor pulmonale*; and
2. to compare the effects of PCV and PSV on lung and heart.

## Methods

Thirty-six Wistar rats were randomly divided into 2 groups. In ELA group, rats received porcine pancreatic elastase (2 IU) intratracheally, once a week, during 4 weeks, whereas SAL group was treated with saline. Lung mechanics and histology, as well as echocardiography were analyzed at 4, 6, 8, and 11 weeks. Additional thirty-two animals were treated with saline (n=16) and elastase (n=16) using the same protocol previously described and randomized according to ventilator strategy: pressure-controlled ventilation (SAL-PCV and ELA-PCV, n=8/each) and pressure-support ventilation (SAL-PSV and ELA-PSV, n=8/each). Tidal volume was kept constant (» 6-8 ml/kg) Lung mechanics and echocardiography were performed at the beginning (Initial) and 4 hours MV (Final), after which lungs were removed for morphometric analysis.

## Results

Compared to SAL group, ELA animals showed increased specific elastance, functional residual capacity, mean alveolar diameter, and right ventricle area at 6 (61%, 37%, 64%, and 8%, respectively) and 8 weeks (37%, 37%, 40%, and 16%, respectively). At 11 weeks, no significant changes were observed in lung mechanics but Lm (53%) and right ventricle area (20%) remained elevated. Based on these data we opted to compare the mechanical ventilation strategies at 8 weeks. Mean airway pressure (Pmean,_aw_) was lower in ELA-PSV compared to ELA-PCV at the Final (p< 0.01). Transpulmonary peak pressure (Ppeak,L) was more reduced in ELA-PSV in Final compared to Initial (p< 0.05). PCV resulted in greater areas of alveolar hyperinflation compared to PSV (p< 0.05). The ratio between the pulmonary artery acceleration time and the pulmonary artery ejection time (PAT/PET) increased in ELA-PSV (16%) along time, but not in ELA-PCV, suggesting reduction in pulmonary arterial hypertension.

## Conclusion

In the current elastase-induced emphysema, we found that: 1) 8 weeks was the time associated to lung mechanical and histological changes and cardiac impairment that resemble human emphysema; and 2) PSV attenuated lung mechanics, hyperinflation, and cardiovascular dysfunction compared to PCV.

## Supported by

CNPq, FAPERJ, CAPES, PRONEX, INCT-INOFAR

